# Insights From Staff and Faculty Focus Groups to Advance Age-Inclusive Professional Development

**DOI:** 10.1093/geroni/igaf122.2145

**Published:** 2025-12-31

**Authors:** Rebekah Perkins, Joshua Seabury, Katarina Friberg-Felsted, Kara Dassel, Sara Hart, Jacqueline Eaton

**Affiliations:** University of Utah, Salt Lake City, Utah, United States; University of Utah, Salt Lake City, Utah, United States; University of Utah, Salt Lake City, Utah, United States; University of Utah, Salt Lake City, Utah, United States; University of Utah, Salt Lake City, Utah, United States; University of Utah, Salt Lake City, Utah, United States

## Abstract

Ensuring age-inclusivity in higher education is essential for fostering professional development among staff and faculty, supporting student success, and strengthening workforce sustainability. However, higher education remains largely age-stratified, often overlooking the professional development needs of a multigenerational workforce and student body. Applying a lifespan lens, the purpose of this presentation is to describe the process of planning and implementing focus groups, within a College of Nursing, to understand faculty, staff, and student needs specific to age inclusivity. Baseline results from the Age-Friendly Inventory and Campus Climate Survey (ICCS) (n = 240) and two student focus groups (n = 13) exploring non-traditional (25+) student experiences informed the focus group interview guide. Three focus groups with staff and faculty (n = 15) and one validation focus group with staff leadership (n = 8) were conducted to gather feedback on ICCS and student focus group results. Analyzed using Braun and Clarke’s thematic analysis, six themes were identified: Mentorship Needs emphasized structured onboarding support. Support Resources highlighted gaps in student success strategies. Succession Planning addressed knowledge transfer. Intergroup Collaboration revealed a need to strengthen engagement across faculty, staff, and students. Healthy Aging Representation emphasized work-life balance considerations. Policy Considerations emphasized institutional policies with a multigenerational workforce Findings highlight the need for lifespan awareness in professional development. Addressing these training needs supports student retention, staff and faculty engagement, and institutional sustainability. Informing the Champions Across the Lifespan training initiative, staff and faculty will apply an age-inclusive lens, enhancing educational excellence and professional growth.

